# ITPR3 facilitates tumor growth, metastasis and stemness by inducing the NF-ĸB/CD44 pathway in urinary bladder carcinoma

**DOI:** 10.1186/s13046-021-01866-1

**Published:** 2021-02-11

**Authors:** Mengzhao Zhang, Lu Wang, Yangyang Yue, Lu Zhang, Tianjie Liu, Minxuan Jing, Xiao Liang, Minghai Ma, Shan Xu, Ke Wang, Xinyang Wang, Jinhai Fan

**Affiliations:** 1 Department of Urology, The First Affiliated Hospital of Xi’an Jiaotong University, #277 Yanta West Road, Xi’an, 710061 China; 2 Department of Hepatobiliary Surgery, First Affiliated Hospital of Xi’an Jiaotong University, Xi’an, China; 3 Oncology Research Lab, Key Laboratory of Environment and Genes Related to Diseases, Ministry of education, Xi’an, China

**Keywords:** ITPR3, Proliferation, Metastasis, Cancer stem cell, Bladder cancer, Epithelial–mesenchymal transition

## Abstract

**Background:**

Bladder carcinoma is one of the most common urological cancers. ITPR3, as a ubiquitous endoplasmic reticulum calcium channel protein, was reported to be involved in the development and progression of various types of cancer. However, the potential roles and molecular mechanism of ITPR3 in bladder cancer are still unclear. Herein, we elucidated a novel role of ITPR3 in regulating the proliferation, metastasis, and stemness of bladder cancer cells.

**Methods:**

The expression of ITPR3 in bladder cancer was analyzed using public databases and bladder cancer tissue microarrays. To demonstrate the role of ITPR3 in regulating the NF-ĸB/CD44 pathway and the progression of bladder cancer, a series of molecular biology and biochemistry methods was performed on clinical tissues, along with in vivo and in vitro experiments. The methods used included western blot assay, quantitative RT-PCR assay, immunofluorescence assay, immunohistochemistry (IHC) assays, wound healing assay, Transwell assay, colony formation assay, tumorsphere formation assay, cell flow cytometry analysis, EdU assay, MTT assay, cell transfection, bisulfite sequencing PCR (BSP), a xenograft tumor model and a tail vein cancer metastasis model.

**Results:**

Higher ITPR3 expression was found in bladder cancer tissues and bladder cancer cells compared with the corresponding normal peritumor tissues and SV-HUC-1 cells, which was attributed to demethylation in the ITPR3 promoter region. ITPR3 promoted the proliferation of bladder cancer by accelerating cell cycle transformation and promoted local invasion and distant metastasis by inducing epithelial-to-mesenchymal transition (EMT). Meanwhile, ITPR3 maintained the cancer stemness phenotype by regulating CD44 expression. NF-κB, which is upstream of CD44, also played a critical role in this process.

**Conclusions:**

Our study clarifies that ITPR3 serves as an oncogene in bladder cancer cells and represents a novel candidate for bladder cancer diagnosis and treatment.

**Supplementary Information:**

The online version contains supplementary material available at 10.1186/s13046-021-01866-1.

## Background

Bladder cancer (BCa) is one of the most common urological malignancies in developing countries and the second most common urological malignancy in developed countries, with an estimated 900,000 new cases and 250,000 deaths due to bladder cancer worldwide each year [1]. Most bladder cancers are transitional cell carcinomas, with more than 70% of new patients having non-muscle-invasive BCa (stage Tis, Ta, T1) and less than 30% having muscle-invasive BCa (stage T2, T3, T4). Approximately 10–30% of non-muscle-invasive BCa will eventually progress to muscle-invasive BCa, and even metastatic bladder cancer patients and 50% of newly diagnosed bladder cancer patients will experience disease relapse, thus leading to distant metastasis and poor prognoses [2, 3]. As is well-known, bladder cancer characterized by high recurrence and distant metastasis is usually accompanied by a poor prognosis [4]. Therefore, there is an urgent need to understand the biological and molecular mechanisms underlying BCa malignant proliferation and distant metastasis.

ITPR3, which is also known as Type 3 inositol 1,4,5-trisphosphate receptor, is one of three highly homologous isoforms of ITPRs (inositol 1,4,5 trisphosphate receptors) (ITPR1, ITPR2 and ITPR3), which are ubiquitous Ca^2+^ channels located at mitochondria/endoplasmic reticulum (ER) contact sites that mediate Ca ^2+^ release from the ER to mitochondria [5]. Intracellular Ca^2+^, which acts as a second messenger, can regulate gene transcription, cell proliferation, migration, invasion, and cell death. Targeting specific calcium signaling has become an emerging research area for human cancer therapy [6]. ITPRs, as the most extensive Ca^2+^ channels, are receiving increasing attention, among which ITPR3 is the principal isoform [7]. It has been reported that ITPRs are emerging as critical sites for the regulation of cell death and survival processes, dysregulation of which leads to oncogenesis. ITPR3, as the principal isoform of ITPRs, was considered to have an anticancer effect by promoting proapoptotic mitochondrial Ca2+ transfer [8]. However, three recent studies have found that cancer cells can adapt to the upregulation of ITPR3 and depend on it, thus driving oncogenesis and malignant cell transformation [6, 7, 9]. Until now, the effect of ITPR3 in human cancer has remained controversial and confusing. However, as a calcium channel regulatory protein between the ER and mitochondria, the biological effects and expression of ITPR3 in human cancer development and progression are still unclear, especially in BCa.

In this study, we demonstrated for the first time that ITPR3 is abnormally overexpressed in bladder cancer tissues compared with normal tissues. In addition, we found that ITPR3 accelerated the malignant progression of bladder cancer by promoting proliferation, metastasis, and cancer stemness in vitro and in vivo, and in this process, the role of the NF-κB/CD44 signaling pathway could not be neglected. The revelations of the molecular mechanism and function of ITPR3 will be helpful in understanding the malignant progression of bladder cancer, in which ITPR3 might be a novel target for the diagnosis and treatment of bladder cancer, especially metastatic bladder cancer, in the future.

## Materials and methods

### Cell culture

The human BCa cell lines T24, UM-UC-3, 5637, and 253 J and the normal urothelium cell line SV-HUC-1, which is an SV-40 immortalized human uroepithelial cell line, were purchased from the American Type Culture Collection (ATCC, Manassas, VA, USA). The cell lines mentioned above (except for 5637) were maintained in DMEM culture medium, and 5637 cells were cultured in RPMI-1640 medium supplemented with 10% FBS (Gibco, Grand Island, NY, USA) and 1% antibiotic-antimycotic (HyClone Laboratories, Logan, UT) at 37 °C aired with 5% CO_2_ and 95% humidity in a cell incubator. The T24-L subcellular line containing luciferase was generated as described in previous research [10] and cultured in DMEM under the same conditions described above. All cell lines used in the research were at early passages.

### Chemicals and reagents

MTT purchased from Sigma-Aldrich Co. (St. Louis, MO, USA) was dissolved in 5 mg/ml with PBS (phosphate-buffered normal saline). Transwell chambers with 8 μM pore polycarbonate membrane filters were purchased from Millipore (Darmstadt, Germany). TGF-β (10 ng/ml), EGF (20 ng/ml), and bFGF (10 ng/ml) were purchased from PeproTech (New Jersey, USA). Tumor necrosis factor-alpha (TNF-α, Invitrogen, Carlsbad, CA, USA), as an activator of the NF-ĸB signaling pathway, was obtained from Invitrogen, and the final concentration was 10 ng/ml in culture medium maintained for 24 h in subsequent experiments. 5-Aza-2′-deoxycytidine used to induce demethylation in bladder cancer cells at a concentration of 2.5 μM or 5 μM for 72 h was purchased from TargetMol (Boston, USA).

### MTT assay

Bladder cancer cells in the logarithmic growth phase were treated with the indicated treatment, and then the viability of the bladder cancer cells was detected by a 3-(4,5-dimethylthiazol-2-yl)-2,5-diphenyltetrazolium bromide (MTT) (Sigma, St. Louis, MO, USA) assay. The specific procedure was as follows. Bladder cancer cells were suspended at a concentration of 20,000 cells per milliliter and seeded into 96-well plates at 200 μl per well. After incubation in the cell incubator for a specific time, 20 μl MTT was added to 180 μl complete medium per well. Four hours later, the absorbance was measured at 490 nm by an ELISA reader (Bio-Rad, Hercules, CA, USA). The data are the result of three independent experiments.

### Bisulfite sequencing PCR (BSP)

CpG islands are closely related to the methylation level of genes. CpG islands in the promoter region of ITPR3 were predicted by MethPrimer (http://www.urogene.org/methprimer/). Genomic DNA (1 μg) from bladder cancer cells and SV-HUC-1 cells was modified and purified with sodium bisulfite using the EpiTect Bisulfite Kit (Qiagen, cat: 59824 Hilden, Germany). Bisulfite genomic sequencing was used to analyze the methylated CpGs in the ITPR3 promoter, and the primers used for bisulfite sequencing PCR were ITPR3 F: 5′-ATTTGTATGTGTGTGGTGGTTT-3′ (sense) and 5′-TAAAACCATTAACRAAACCCTC-3′ (antisense). Amplified bisulfate PCR products were cloned into the pMD18-T simple vector (Takara, Dalian, China). Ten bacterial colonies containing 43 methylation sites were selected for sequencing at Sangon Biotech (Shanghai, China).

### Clinical specimens, tissue chip and immunohistochemistry (IHC) assays

BCa patients’ tissues and its corresponding normal tissues (*n* = 11) were obtained from the Department of Urology, The First Affiliated Hospital of Xi’an Jiaotong University, Xi’an, China. All clinical samples used in the research were approved by the Ethical Committee of the Hospital, and informed consent was obtained from all the BCa patients (*n*=11) who participated in this study. In order to further investigate the expression of ITPR3 in BCa and matched normal tissues, we purchased a BCa tissue chip (Cat No. HBlaU060CS02) from Outdo Biotech Co., Ltd. (Shanghai, China) containing 30 individual BCa patient tissues, with each adjacent noncancerous tissue placed beside its matched cancer tissue. The tissue microarray slide was deparaffinized at 60 °C for 4 h, rehydrated in graded solutions of ethanol (100, 95, 80, 70 and 50%) for 3 min, and then subjected to antigen repair at 121 °C for 5 min and endogenous enzyme blocking at room temperature for 60 min. After blocking with 5% BSA for 1 h, the tissue chip was incubated with the primary antibody at 4 °C overnight. On the second day, after incubation with Envision-HRP secondary antibody for 30 min at room temperature, the images were detected by an Olympus BX51 microscope (Olympus Corporation, Tokyo, Japan) after staining with a DAB kit according to the manufacturer’s protocols. The staining images were assessed according to the staining intensity (0, 1, 2+, 3+) and the percentage of positive cells (0 (0%), 1 (1–25%), 2 (26–50%), 3 (51–75%) and 4 (76–100%)). The degree of protein expression in the image was evaluated by the combination of the staining score and the percentage of positive cells and presented in the way of (0 score), weak (1–4 score), moderate (5–8 score) and strong (9–12 score).

### Plasmid transfection and lentiviral infection

The lentiviral vector (Psi-LVRU6GP), which encodes short hairpin RNA (shRNA) targeting ITPR3 and scramble control shRNA, was constructed by GeneCopoeia (Guangzhou, China). The lentiviruses were packaged to improve the transfection efficiency according to the procedure described previously [11]. Lentivirus-overexpressing CD44 and empty lentivirus vectors were obtained from GeneChem Company (Shanghai, China). The cells were used for subsequent experiments after lentivirus transfection for 48 h with 8 μg/ml polybrene existed.

### Wound healing assay

The wound healing assay was executed to assess the migration ability of 5637 and 253 J cells under specific conditions. The cells were seeded into 6-well plates with the marker lines across the bottom side and scratched with a 200 μl pipette tip to mark the distance when the cell density reached almost 100%. Serum-free medium was added to the 6-well plates, and images were captured by an inverted microscope (Olympus, Tokyo, Japan) every 24 h until the scratch was almost closed. This experiment was repeated in triplicate.

### Transwell assay

Migration and invasion assays were performed via Boyden chambers with an 8-μm pore size (Millipore, Germany) to evaluate the migration and invasion abilities of bladder cancer cells under given conditions. For the migration assay, chambers plated into 24-well plates were seeded with 4 × 10^4^ 5637 and 2 × 10^4^ 253 J cells suspended in 200 μl serum-free culture medium in the upper chamber without Matrigel, and 800 μl medium with 10% FBS was added to the lower chamber for 24 h. For the invasion assay, 8 × 10^4^ 5637 and 4 × 10^4^ 253 J suspended in 200 μl serum-free culture medium were added to the upper chamber with 60 μl Matrigel (Sigma, St. Louis, MO, USA) and incubated in a cell incubator at 37 °C for 4 h, and 800 μl medium with 10% FBS was added to the lower chamber for 48 h. After washing with PBS three times, fixing with 4% paraformaldehyde for 15 min and staining with 0.1% crystal violet for 5 min, the visible cells were observed and counted under an inverted light microscope (magnification, × 100) in three random fields for each chamber. The experiment was executed in triplicate.

### Immunofluorescence assay

Immunofluorescence assays were conducted as previously described [12]. Briefly, BCa cells were fixed with 4% paraformaldehyde, permeabilized with 0.5% Triton-100 in PBS, blocked with 5% BSA (bovine serum albumin) and incubated with the indicated primary antibodies dissolved in PBS 1:200 at 4 °C overnight. After incubation with the fluorescent secondary antibody at room temperature for 1 h, staining with DAPI for 5 min in the dark and sealing with glycerin, the stained images were captured under a positive fluorescence microscope (Olympus, Tokyo, Japan). The experiments were performed at least in triplicate.

### Quantitative RT-PCR

The total RNA was isolated from BCa cells with RNAfast 200 reagents (Feijie Biotechnology, Shanghai, China) according to the manufacturer’s instructions, quantified by absorbance at 260 nm and reverse-transcribed to complementary DNA using a Prime Script RT-PCR kit (Takara Bio Dalian, China). The cDNA was amplified to detect the expression of specific genes using a CFX96 Real-Time PCR system (Bio-Rad, CA, USA) with SYBR-Green PCR Master Mix (Takara Bio, Dalian, China). Gene-specific primers were as follows: ITPR1, F: GCGGAGGGATCGACAAATGG and R: TGGGACATAGCTTAAAGAGGCA; ITPR2, F: CACCTTGGGGTTAGTGGATGA and R: CTCGGTGTGGTTCCCTTGT; ITPR3, F: CCAAGCAGACTAAGCAGGACA and R: ACACTGCCATACTTCACGACA; and 18S, F: CAGCCACCCGAGATTGAGCA and R: TAGTAGCGACGGGCGGTGTG. Gene mRNA expression levels were assessed by the 2^−ΔΔCt^ method. 18S was used for normalization.

### Western blot assay

After treatment with specific experimental conditions, the total proteins were isolated with RIPA lysis buffer (Catalog Number P0013B, Beyotime, China) with a protease inhibitor, phosphatase inhibitor and 0.1 M PMSF (Catalog Number ST506, Beyotime, China). Nuclear and cytoplasmic proteins were extracted using a Nuclear Extraction Kit (Abcam, ab113474, Shanghai, China). The collection of proteins and western blot assays were performed as previously described [12]. The following antibodies were used in the experiment: ITPR3 (BS72246) used for WB was purchased from Bioworld Technology. Inc., and ITPR3 (ab264282) used for IHC was purchased from Abcam. ITPR1 (19962–1-AP), E2F1 (12171–1-AP), Cyclin D1 (26939–1-AP), Cyclin B1 (55004–1-AP), CDK2 (10122–1-AP), P21 (10355–1-AP), MMP2 (10373–2-AP), MMP9 (10375–2-AP), CD44 (15675–1-AP), P63 (12143–1-AP), OCT4 (11263–1-AP), IkBα (10268–1-AP), and KLF4 (11880–1-AP) were purchased from Proteintech Group. SOX2 (A0561) and β-actin (AC026) were purchased from Abclonal. ITPR2 (DF13336) was obtained from Affinity. E-cadherin (3195), N-cadherin (13116), Vimentin (5741), NF-κB (8242), Phospho-NF-κB (Ser536) (3033), Phospho-IκBα (Ser32) (2859), Snail1 (3879), and Snail 2 (9585) were purchased from CST (Cell Signaling Technology).

### Colony formation assay and tumorsphere formation assay

After treatment with various desired conditions, 5637 and 253 J BCa cells were seeded into 6-well plates at 1000 cells per well and incubated in a cell incubator for seven days with the medium replaced every 48 h. After washing with PBS three times, the cells were fixed with 4% paraformaldehyde for 15 min and stained with 0.1% crystal violet for 5 min to make the cells visible. The clonogenicity was assessed by counting the number of colonies in five random fields at 100X magnification. The experiments were conducted in triplicate. BCa cells were suspended and seeded into 6-well ultralow attachment plates with 3 ml/well cancer stemness medium containing 2xB27, 1xN_2_ supplement, 20 ng/ml EGF (epidermal growth factor) and 10 ng/ml bFGF (basic fibroblast growth factor). After cultivation for two weeks, tumorsphere formation was counted and analyzed by microscopy in five random fields. The experiment was executed in triplicate.

### Cell flow cytometry analysis

Human BCa cell lines 253 J and 5637 were maintained in DMEM or RPMI-1640 medium supplemented with 10% FBS in 6 cm dishes. After treatment with the given conditions, the cells were collected and suspended in PBS, fixed with 70% ice-cold ethanol at 4 °C overnight and then incubated with 50 μg/ml PI (propidium iodide) and 100 μg/ml RNase A (1:1) in PBS at room temperature in the dark for at least 15 min. Finally, a FACSCalibur™ flow cytometer (BD Biosciences, Franklin Lakes, NJ, USA) was used to assess the staining signals of the BCa cells, and Cell Quest software version 3.3 (BD Biosciences) was used to analyze the percentage of cell cycles distributed according the manufacturer’s instructions. The experiment was repeated three times.

### EdU assay

The proliferation ability of BCa cells was detected by EdU assay using the EdU Cell Proliferation Kit with Alexa Fluor 594 (Beyotime, China). Cells were washed with PBS three times and incubated with complete medium with 10 μM EdU in a cell incubator for at least 2 h. Then, the cells were washed with PBS to remove the EdU probe and culture medium, fixed with 4% paraformaldehyde for 10 min at room temperature, and stained with DAPI for 5 min in the dark. The cells were observed under a positive fluorescence microscope (Olympus, Tokyo, Japan) at 100x magnification in five random fields. The staining signals were captured, analyzed, and shown as fold changes compared with the control. The experiment was repeated three times.

### Gene set enrichment analysis

The mRNA expression data of 408 BCa patients were downloaded from The Cancer Genome Atlas (TCGA) database (https://www.cancer.gov/tcga). The ITPR3 high-expression group (top 25%, 102 of 408 patients in TCGA FPKM cohort) and ITPR3 low-expression group (top 25%, 102 of 408 patients in TCGA FPKM cohort) were set up. Gene Set Enrichment Analysis (GSEA) 2.0 software was used to analyze the significantly changed pathways and top 100 altered genes of the two groups after the data were submitted to the software and the hallmark gene sets were selected for analysis (http://software.broadinstitute.org/gsea/msigdb/collections.jsp#H) [13].

### Xenograft tumor model and tail vein cancer metastasis model

All male athymic nude mice used in the study were approved by the Ethical Committee of the First Affiliated Hospital of Medical College, Xi’an Jiaotong University, Xi’an, China. Ten 4-week-old nude mice were randomly separated into two groups with each group containing 5 mice and then injected subcutaneously in both flanks with 100 μl serum-free culture medium containing 50 μl Matrigel and 5 × 10^6^ 5637 cells (shCon or shITPR3) after 7 days of feeding. Then, the mouse weight and tumor size were recorded and measured every three days until the nude mice with tumors were euthanized to harvest the tumors at day 30. The tumors were weighed, measured, and then stained by immunohistochemistry. The formula for calculating the tumor volume was (length × width ^2^) × 0.5. The total protein of the tumors was extracted with RIPA lysis buffer and detected by a western blot assay. A nude mouse tail vein cancer metastasis model was established as previously described [12]. Briefly, ten 4-week-old nude mice were randomly divided into two groups with each group containing 5 mice, and 2 × 10^6^ T24-L BCa cells (shCon or shITPR3) labeled with luciferase suspended in 200 μl serum-free culture medium were injected via the tail vein with an insulin needle after 7 days of feeding. Bioluminescence imaging (BLI) was performed to monitor distant metastasis in the lung and other organs at day 30 after injection with 450 mg/kg D-luciferin (Abcam, ab143655, Shanghai, China). Lungs with distant metastatic foci were obtained from nude mice to observe the number of lung surface metastatic foci and then stained by hematoxylin-eosin (HE) and immunohistochemistry after the mice were euthanized.

### Bioinformatics and statistical analysis

The GSE3167 dataset used to analyze the expression of ITPR3 between tumor and normal tissues and the GDS183 dataset used to detect the expression in different clinical stages were downloaded from NCBI GEO (Gene Expression Omnibus). The expression level of ITPR3 in bladder cancer and normal tissues and the overall survival and disease-free survival related to ITPR3 mRNA expression level were analyzed with GEPIA (http://gepia.cancer-pku.cn/) and cBioPortal (www.cbioportal.org) for The Cancer Genome Atlas (TCGA). ITPR3 mRNA expression in multiple kinds of cancers, including bladder cancer, was analyzed from the Oncomine database (https://www.oncomine.org/). Briefly, the differences between two groups were analyzed by Student’s t-test implemented with GraphPad Prism 7.0 software (GraphPad, CA, USA). The data are presented as the mean ± SD of three independent experiments. *P* < 0.05 indicated statistical significance.

## Results

### ITPR3 was commonly overexpressed in BCa cells and tissues

**Fig. 1:**
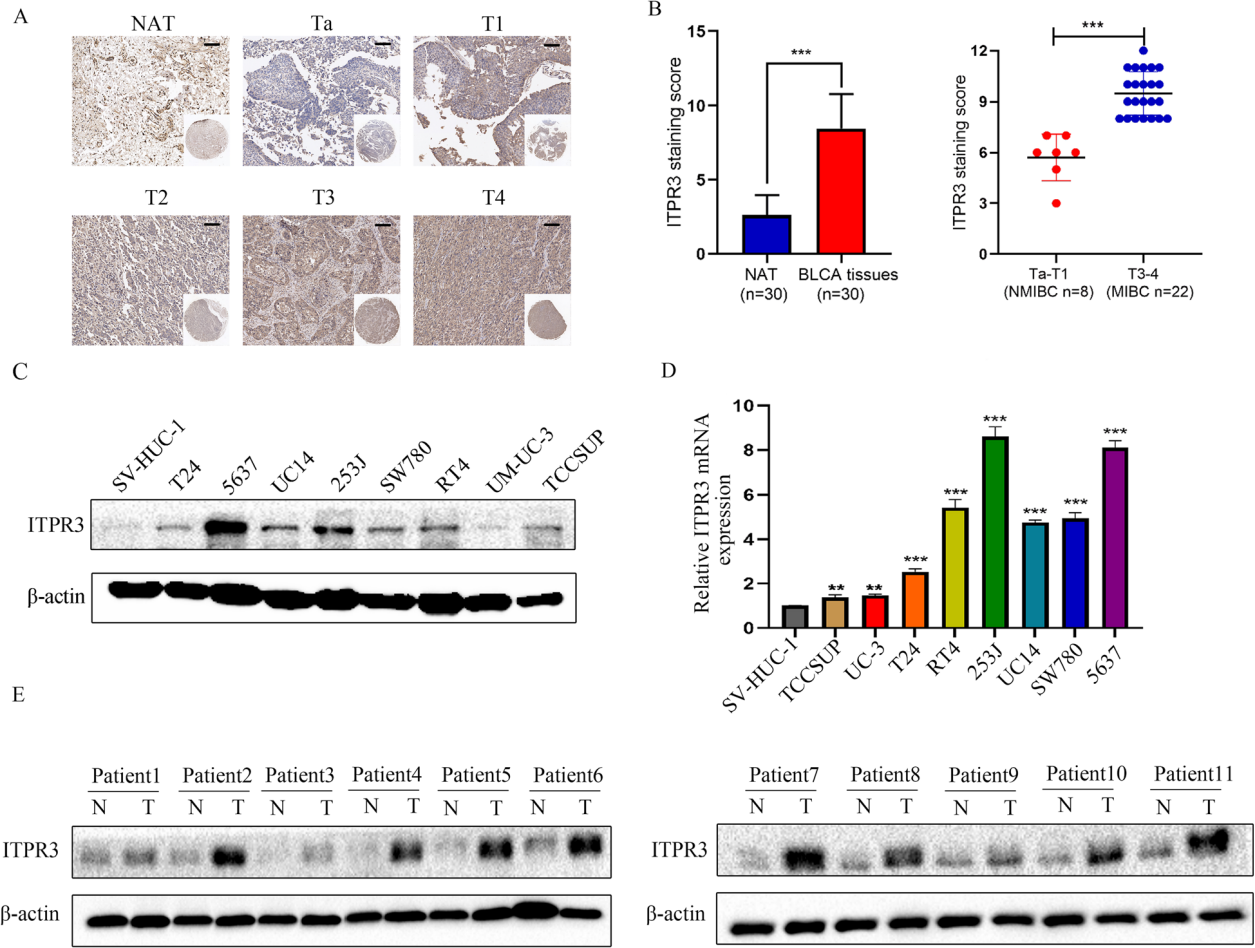
ITPR3 was commonly overexpressed in BCa cells and tissues. **a** Representative pictures of ITPR3 protein expression of different pathologic T stage in bladder cancer tissue chips detected by IHC. **b** Quantification of the ITPR3 protein in bladder cancer and normal peritumor tissues. Scale bar = 100 μm. **c** western blot analysis of ITPR3 expression levels in human bladder cancer cell lines and the normal bladder cell line SV-HUC-1. β-actin was used as the loading control. **d** Quantitative real-time PCR (qRT-PCR) analysis of the mRNA expression levels of ITPR3 in bladder cancer cell lines and the normal bladder cell line SV-HUC-1. 18S was applied as the endogenous control. Data are presented as the mean ± SEM, *n* = 3. **e** ITPR3 protein expression level was analyzed in eleven cases of fresh human bladder cancer tissues and corresponding normal tissues by western blot assay. β-actin was used as internal loading control ^*^p < 0.05; ^**^p < 0.01; ^***^p < 0.001. NAT: normal tissue adjacent to the tumor; Ta-T4: Pathologic T stage Ta, T1–4; NMIBC: non-muscle invasive bladder cancer; MIBC; muscle invasive bladder cancer

To explore the expression pattern of ITPR3 in BCa tissues and cells, immunohistochemical staining was performed on a bladder cancer tissue microarray containing 30 cases of BCa and matched adjacent nontumor tissues. The results showed that ITPR3 was not only significantly upregulated in BCa tissues compared with corresponding normal peritumor tissues but also positively correlated with clinicopathological stages (Ta-T4) and tumor invasion degree (NMIBC and MIBC) (Fig. 1a, b). Then, we further examined ITPR3 expression in bladder cancer cell lines and SV-HUC-1 cells (SV-40 immortalized uroepithelial cells) at both the protein and mRNA levels (Fig. 1c, d) and found that ITPR3 was significantly upregulated in BCa cell lines, especially in 5637 and 253 J cells, compared to SV-HUC-1 cells, which was consistent with the immunohistochemistry (IHC) results. ITPR3 was also upregulated in the BCa tissues compared with its matched normal adjacent tissues, as detected by the western blot assay (Fig. 1e). At the same time, bioinformatics analysis was also used to confirm our conclusion. From the analysis of GEO datasets (GSE3167, GDS183), the GEPIA website (based on the TCGA database) and the TCGA dataset, we found that ITPR3 was overexpressed in bladder cancer and positively correlated with clinical stages, which was also consistent with the result from immunohistochemistry (IHC) (Supplementary Fig. 1. A-C). The results from the analysis of the Oncomine dataset indicated that ITPR3 was widely overexpressed in various human tumors, including bladder cancer, kidney cancer, pancreatic cancer, etc. (Supplementary Fig. 1. D). From the results of Sanchez-Carbayo’s research from the Oncomine website, we also reached the same conclusion that ITPR3 was highly expressed in bladder cancer (Supplementary Fig. 1. E). Although there is considerable evidence that ITPR3 is overexpressed in bladder cancer, we failed to observe a positive relationship between ITPR3 and the prognosis of patients with bladder cancer, including overall survival (OS) or disease-free survival (DFS) (Supplementary Fig. 1. F).

### Significant overexpression of ITPR3 in bladder cancer is attributed to demethylation of its promoter

**Fig. 2:**
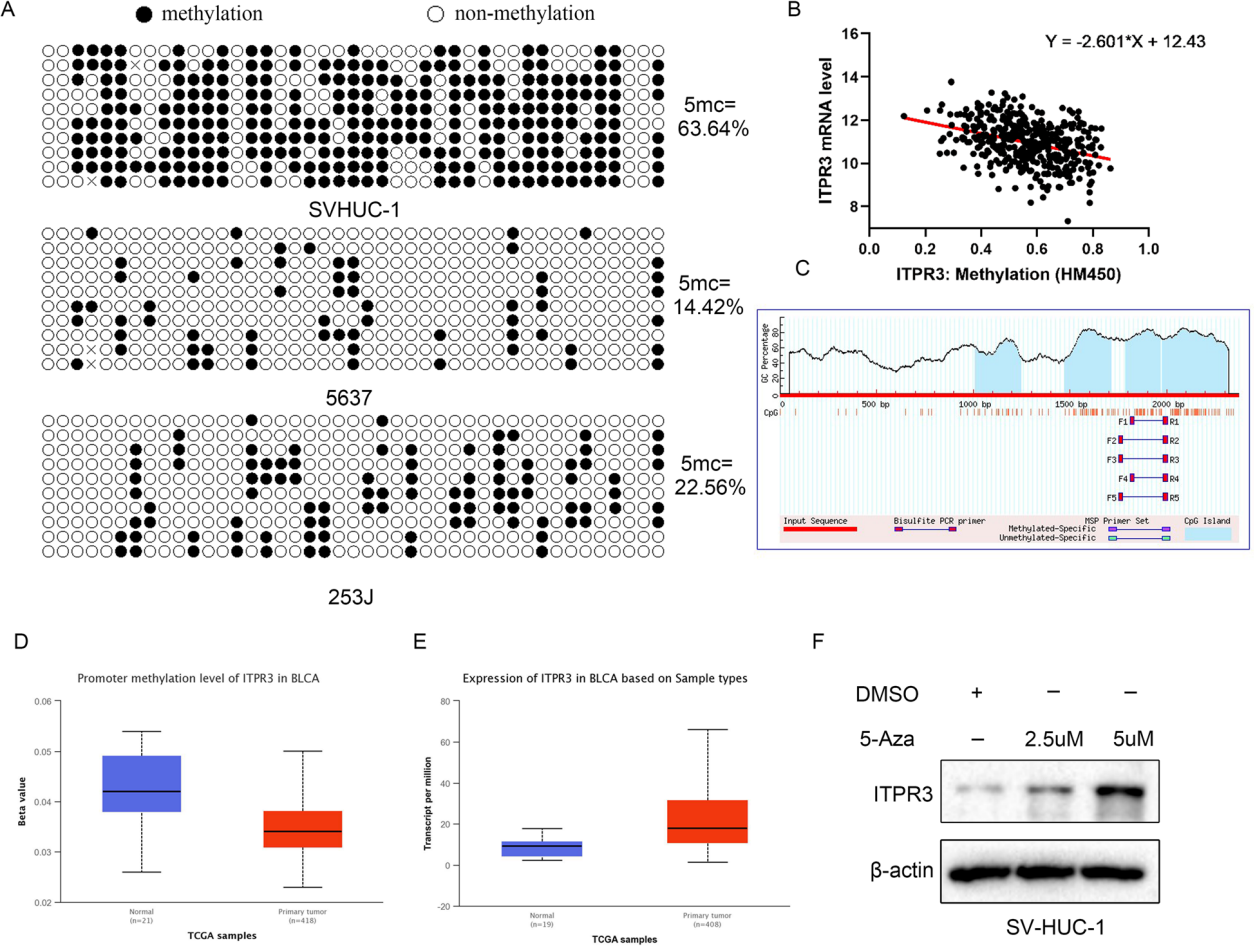
Significant overexpression of ITPR3 in bladder cancer is attributed to demethylation of its promoter. **a** The methylation status of ITPR3 in SV-HUC-1, 5637 and 253 J cells was analyzed by bisulfite sequencing PCR (BSP). The 43 CpG sites in 10 clones were represented. The solid circle represents the methylated cytosine, and the hollow circle represents the unmethylated cytosine. **b** Analysis of the linear correlation between the mRNA level and methylation level of ITPR3 based on TCGA data. **c** Predicted CpG islands in the promoter region of ITPR3. Numbers indicate the positions in bp relative to the transcription start site. The blue regions represent the CpG islands, and the red vertical bars are the CpG loci in these input sequences. The promoter methylation level (**d**) and mRNA expression level (**e**) of ITPR3 in bladder cancer and normal tissue were examined through the UALCAN website based on the TCGA dataset. **f** ITPR3 protein levels were detected by a western blot assay in SV-HUC-1 cells after treatment with different doses of 5-Aza (0 μM, 2.5 μM and 5 μM) for 72 h. β-actin was used as an internal loading control

The methylation status of ITPR3 in SV-HUC-1, 5637 and 253 J cells was analyzed by bisulfite sequencing PCR (BSP) and the representative images were shown in Fig. 2a. The methylation level of ITPR3 in SVHUC-1 cells was much higher than that in bladder cancer cells 5637 and 253 J. Meanwhile, we also found that there was a negative correlation between the methylation level of ITPR3 and its mRNA expression level (Fig. 2b). CpG islands in the promoter region of ITPR3 was predicted by MethPrimer 2.0 and there was four CpG island in the promoter including three CpG islands longer than 200 bp (Fig. 2c). Through UALCAN TOOL, we found ITPR3 was in high expression in bladder cancer while the methylation level of ITPR3 was in low level (Fig. 2d, e). With the treatment of increasing dose of 5-Aza in the normal bladder cell line SVHUC-1, the protein level of ITPR3 was upregulation in a dose-dependent manner (Fig. 2f). The above results fully indicated that the high expression of ITPR3 was caused by demethylation of its promoter region.

### ITPR3 inhibition decreases BCa cell proliferation in vitro

**Fig. 3:**
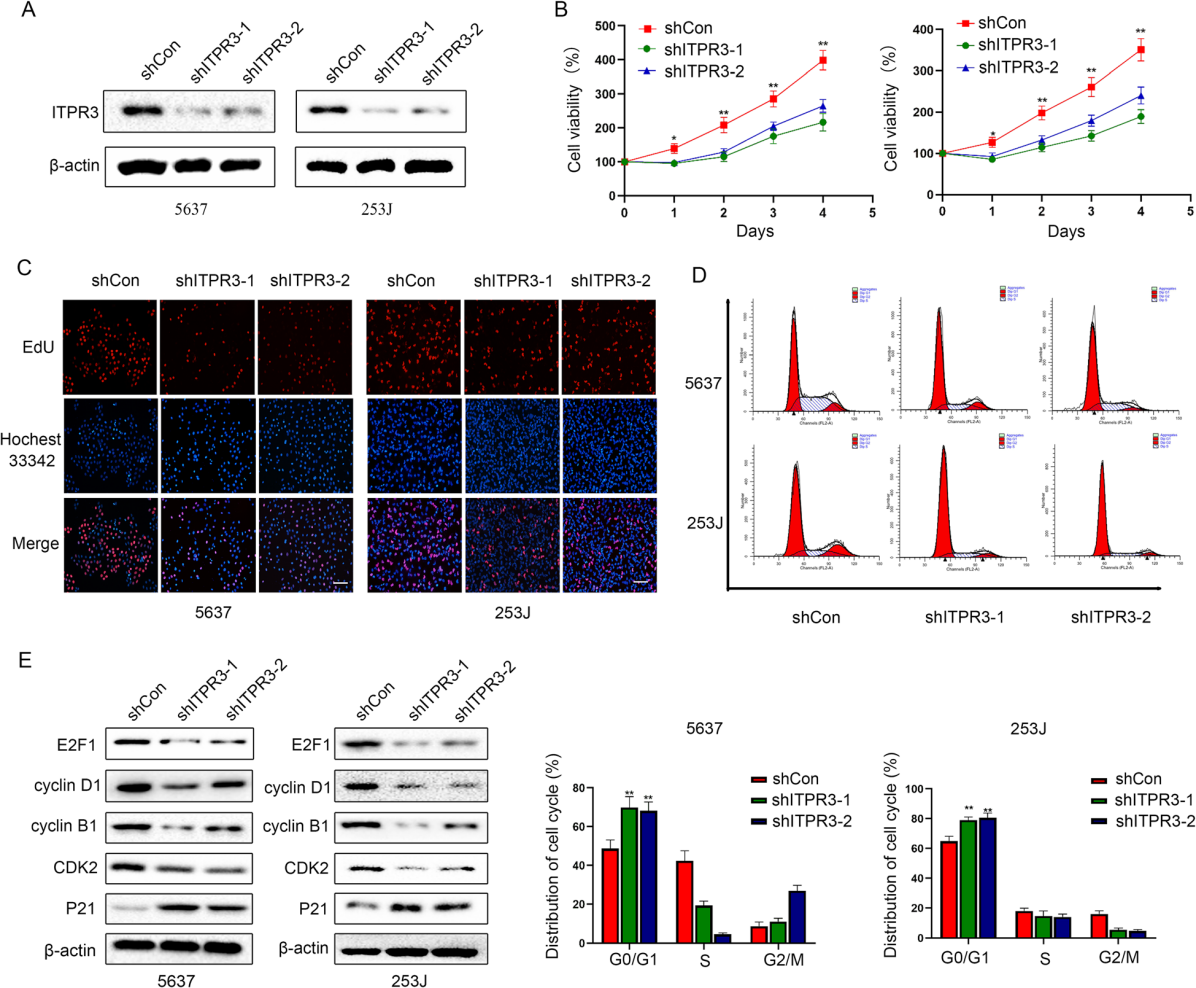
ITPR3 inhibition decreases BCa cell proliferation in vitro. **a** The efficiency of ITPR3 knockdown was verified by western blotting analysis in 5637 or 253 J cell lines transfected with ITPR3 shRNAs and shControl. **b** Cell viability was detected by an MTT assay with treatment as indicated. **c** An EdU incorporation assay for staining proliferating cells (red indicates EdU-incorporated cells; blue indicates nuclear staining with Hoechst 33342) was conducted in 5637 and 253 J cells with treatment as indicated. Scale bars, 100 μm. **d** The cell cycle distribution was analyzed by flow cytometry. Quantitative analysis of the results is shown below. **e** Cell cycle-related proteins were detected by western blotting using the indicated antibodies. The images show the changes in the proteins E2F1, P21, Cyclin D1, Cyclin B1 and CDK2 in 5637 and 253 J cells. β-actin was used as a loading control. ^*^P < 0.05, ^**^P < 0.01

The expression of ITPR3 was knocked down by shRNA in 5637 and 253 J cells with relatively high ITPR3 expression to investigate the function of ITPR3 in bladder cancer, and the inefficiency of knockdown was detected by a western blot assay (Fig. 3a). Stably transfected 5637 and 253 J shITPR3 cells were used for subsequent experiments. The MTT assay revealed that ITPR3 inhibition suppressed the proliferation ability of BCa cells (5637 and 253 J) in a time-dependent manner (Fig. 3b). DNA replication activity, which is usually considered to represent the proliferation capacity, was detected by an EdU staining assay. As is shown in Fig. 2c, ITPR3 suppression significantly reduced the EdU staining signals in 5637 and 253 J cells (Fig. 3c). A similar result was also observed in flow cytometry analysis, in which cell cycle arrest in the G0/G1 phase was observed after ITPR3 knockdown (Fig. 3d). Meanwhile, the expression of cell cycle-related genes such as E2F1, Cyclin D1, Cyclin B1, and CDK2 was also decreased after ITPR3 knockdown while P21, as a classical tumor suppressor gene was upregulated (Fig. 3e). All the data indicated that ITPR3 loss suppressed BCa cell proliferation capacity.

### ITPR3 loss inhibits the migration and invasiveness of bladder cancer cells in vitro

**Fig. 4:**
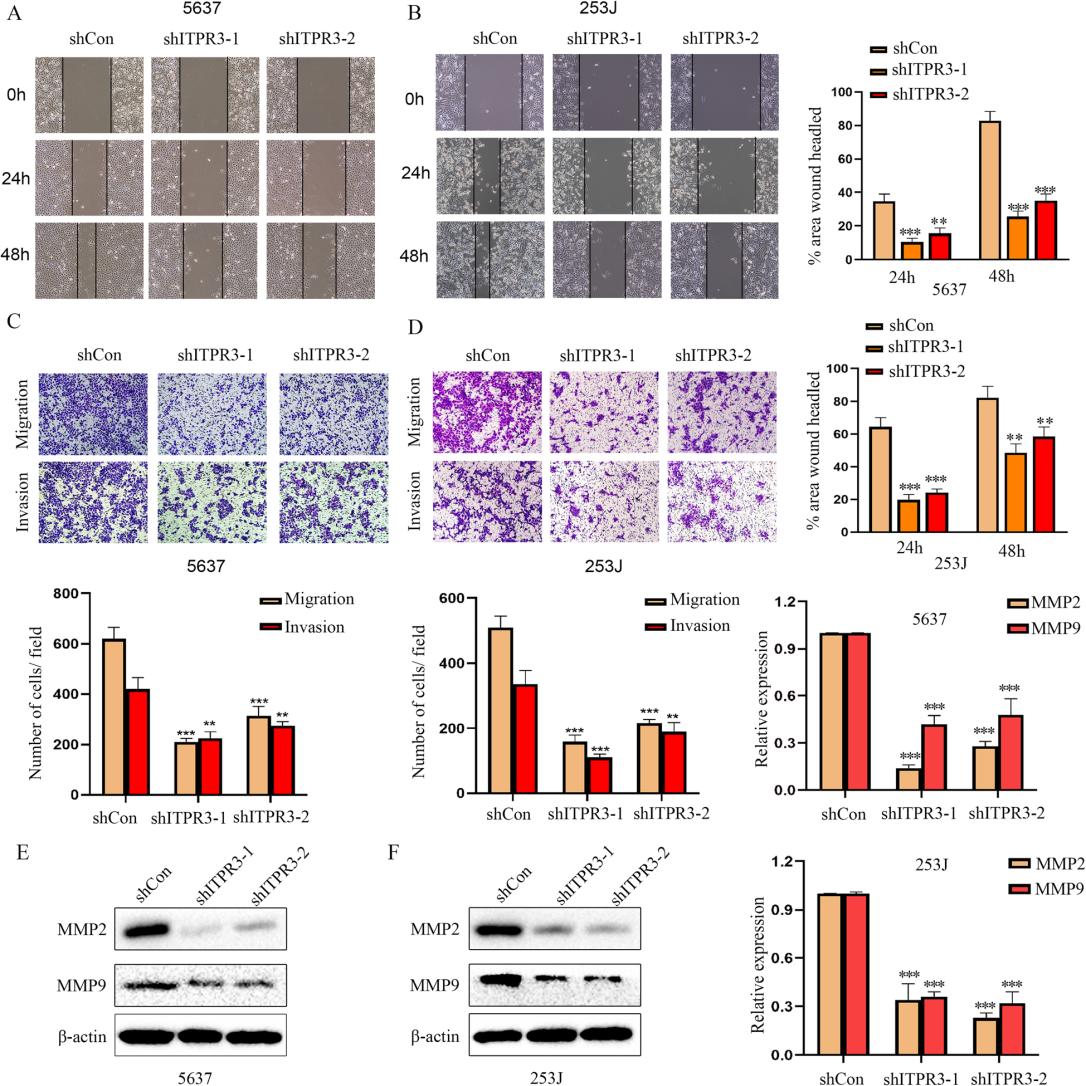
ITPR3 loss inhibits the migration and invasiveness of bladder cancer cells in vitro. Representative images of wound healing in 5637 (**a**) and 253 J (**b**) shITPR3 or shCon cells. Quantitative data are shown on the right. **b** Cell migration and invasion ability was assessed by the Transwell assay with or without Matrigel in 5637 (**c**) and 253 J (**d**) shITPR3 or shCon cells. Quantification analysis is shown below. MMP2 and MMP9 expression was detected by a western blot assay in 5637 (**e**) and 253 J (**f**) shITPR3 or shCon cells. The bar chart represents the ratios of MMP2 and MMP9 relative to β-actin (as a loading control) and is shown on the right. ^**^p < 0.01; ^***^p < 0.001

Sustained proliferation and a high invasion and metastasis capacity are the main manifestations of the malignant progression of tumors [14, 15]. We have demonstrated that ITPR3 can promote cell proliferation by accelerating cell cycle transformation. Furthermore, to investigate the potential role of ITPR3 in the migration and invasion capacity of bladder cancer cells, wound healing assays and transwell assays were conducted simultaneously after ITPR3 knockdown. The results obtained suggest that ITPR3 silencing inhibited the wound healing rate compared with that in the control 5637 and 253 J cells (Fig. 4a, b). Similar results were also observed in the migration assay. A Matrigel invasion assay was conducted to assess the distant metastatic capacity of bladder cancer cells, and ITPR3 inhibition also suppressed the invasion ability of BCa cells (Fig. 4c, d). Matrix metalloproteinases, which belong to a large family of proteases, have been demonstrated to play vital roles in tissue remodeling and cancer progression and metastasis, of which MMP2 and MMP9 are the most important [16, 17]. We found that the MMP2 and MMP9 protein levels as detected by western blot assay were suppressed in 5637 and 253 J cells after ITPR3 knockdown (Fig. 4e, f). All these data suggested that ITPR3 might act as an important factor in bladder cancer metastasis.

### ITPR3 inhibition blocks epithelial-mesenchymal transition in bladder cancer cells

**Fig. 5:**
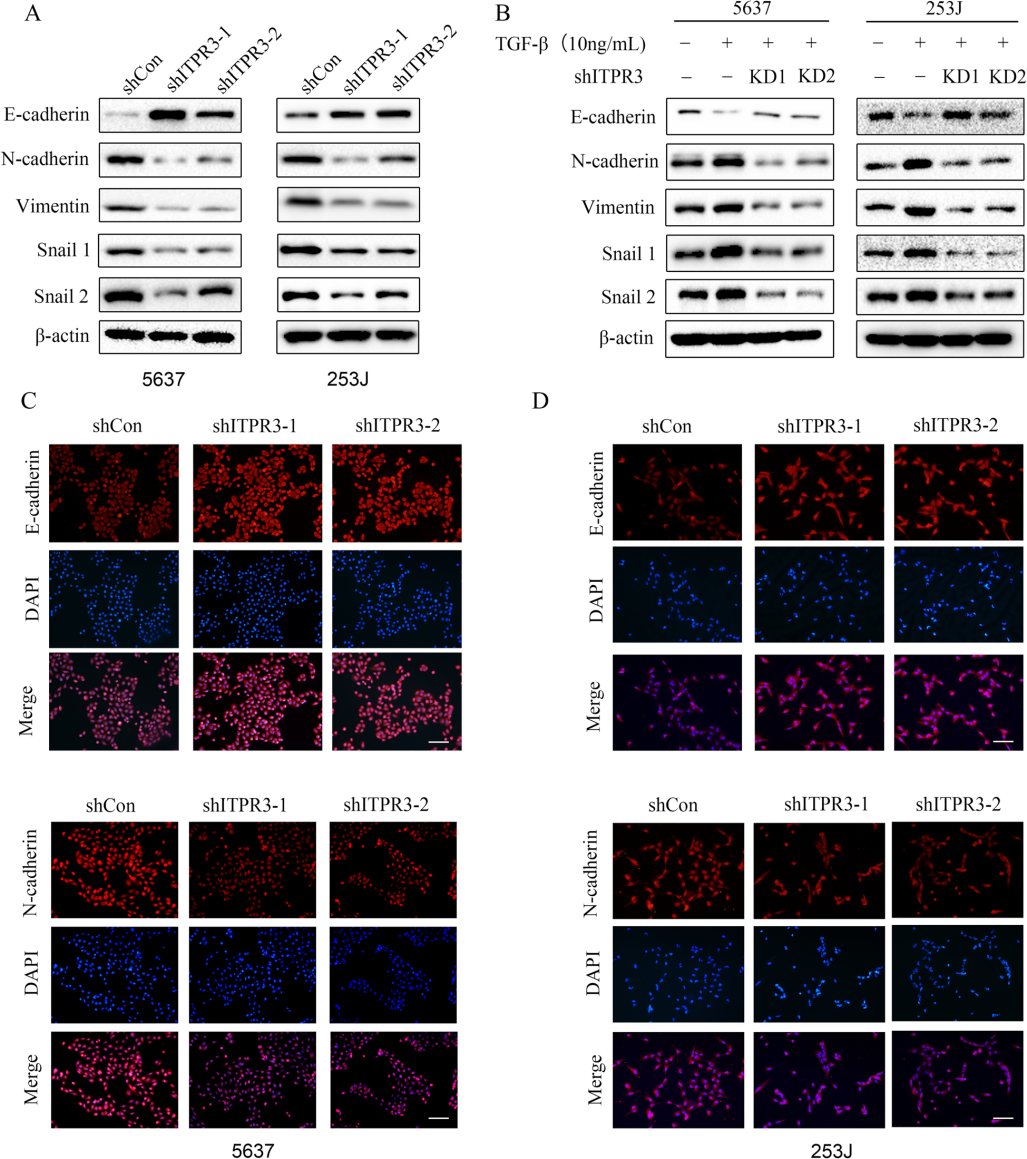
ITPR3 inhibition blocks epithelial-mesenchymal transition in bladder cancer cells. **a** The mesenchymal markers N-cadherin and vimentin, the epithelial marker E-cadherin, and the transcription factors snail 1 and snail 2 were detected by western blotting after ITPR3 knockdown in 5637 and 253 J cells. β-actin served as an internal control. **b** The expression of E-cadherin, N-cadherin, Vimentin, Snail 1, and Snail 2 was detected by western blotting after treatment with TGF-β (10 ng/mL) in 5637 and 253 J cells transfected with ITPR3 shRNA or shCon. β-actin served as an internal control. The results of immunofluorescence assays for E-cadherin and N-cadherin expression were also determined in 5637 (**c**) and 253 J (**d**) cells transfected with ITPR3 shRNA or shCon. Scale bars, 100 μm

Epithelial-mesenchymal transformation (EMT) can result in morphological changes from polygonal to spindle-shaped, which makes cells more susceptible to distant metastasis. EMT is also the leading cause of distant metastasis, which can lead to poor prognoses of cancers [18, 19]. Until now, the potential effect of ITPR3 on EMT has not yet been reported. As shown in Fig. 5a, ITPR3 depletion blocked EMT by simultaneously upregulated an epithelial marker (E-cadherin) and downregulated mesenchymal markers (N-cadherin, Vimentin, Snail 1, and Snail 2) in 5637 and 253 J cells. TGF-β (transforming growth factor-β), acting as a classical activator of EMT, was used to assess the potential role of ITPR3 in EMT. The results revealed that ITPR3 depletion blocked the activation of EMT induced by TGF-β (Fig. 5b). Similar results were also observed in the immunofluorescence assay, in which E-cadherin was upregulated and N-cadherin was downregulated in 5637 and 253 J cells after ITPR3 suppression (Fig. 5c, d). These results suggested that ITPR3 knockdown blocked epithelial-to-mesenchymal transition in bladder cancer cells.

### ITPR3 knockdown suppresses the stemness properties of bladder cancer cells

**Fig. 6:**
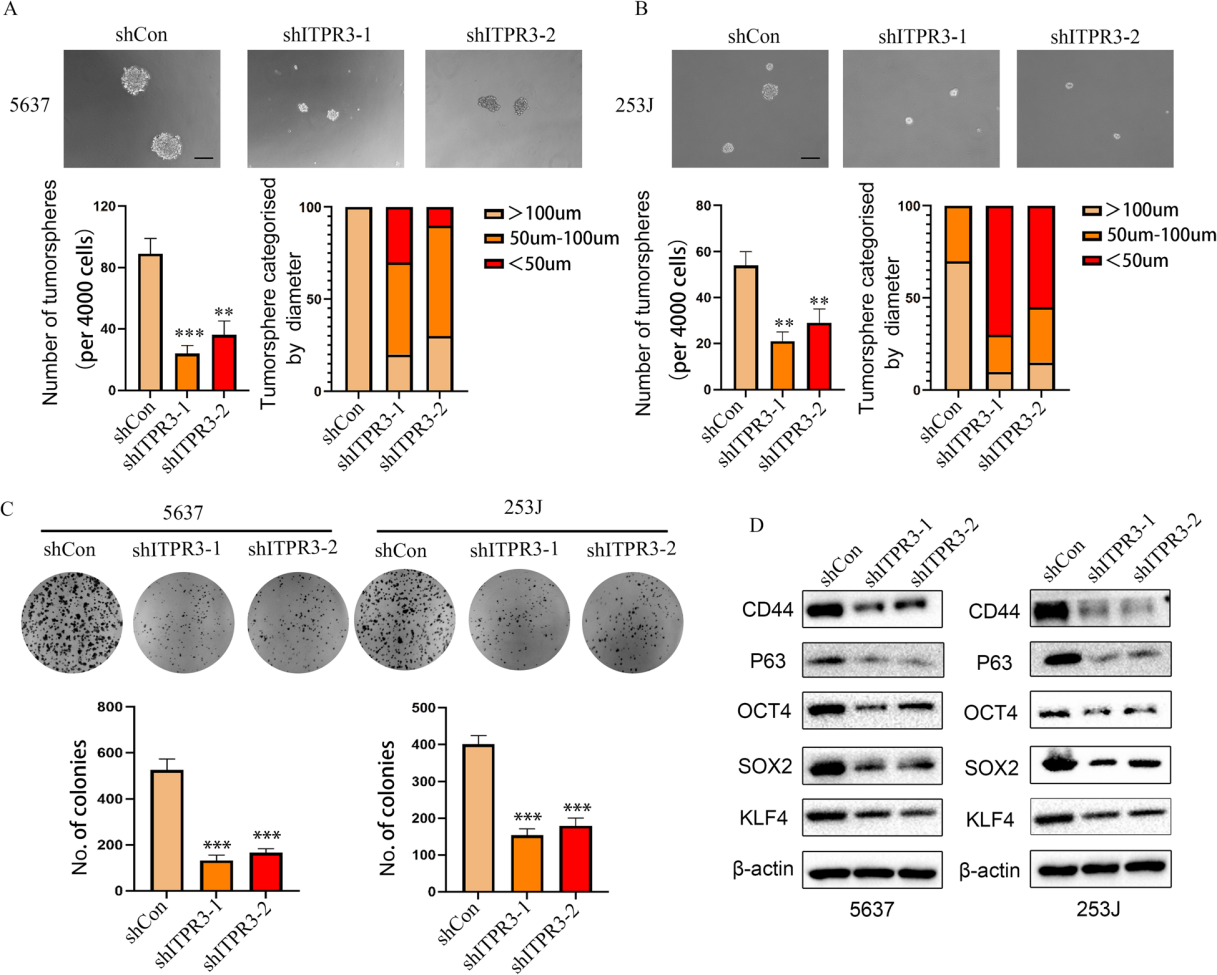
ITPR3 knockdown suppresses the stemness properties of bladder cancer cells. **a**, **b** The cancer stemness of 5637 and 253 J cells transfected with shITPR3 or shCon was evaluated by a tumorsphere assay. The number and diameter were also quantified and plotted. The scale bar represents 100 μm. **c** A colony formation assay was conducted in 5637 and 253 J cells transfected with shITPR3 or shCon, and the colony number was counted and plotted. Quantification analysis was shown below. **d** CSC-related markers, such as CD44, OCT4, SOX2, P63 and KLF4, were examined in 5637 and 253 J cells transfected with ITPR3 shRNA or shCon. β-actin was used as a loading control. ^**^p < 0.05, ^***^p < 0.001

An increasing number of studies have confirmed that cancer stem cells (CSCs) play a vital role in promoting tumor development, relapse, and metastasis and have intrinsic self-renewal characteristics and tumorigenic properties. CSCs might be the root cause of malignant progression and resistance to cancer therapy [20, 21]. Therefore, inhibition of cancer stemness may contribute to the development of clinical strategies and may provide a new direction for the future treatment of cancers [22]. Tumorsphere formation assays and colony formation assays were conducted to evaluate the potential effect of ITPR3 on cancer stemness properties. The results showed that tumorsphere and colony formation abilities were suppressed after ITPR3 knockdown (Fig. 6a-c). Consistently, CSC markers such as CD44, P63, OCT4, SOX2 and KLF4 were also decreased in shITPR3 cells compared with shCon cells (Fig. 6d). These results suggested that ITPR3 inhibition impaired the CSC phenotype in BCa cells.

### ITPR3-mediated NF-ĸB/CD44 signaling in BCa cells

**Fig. 7:**
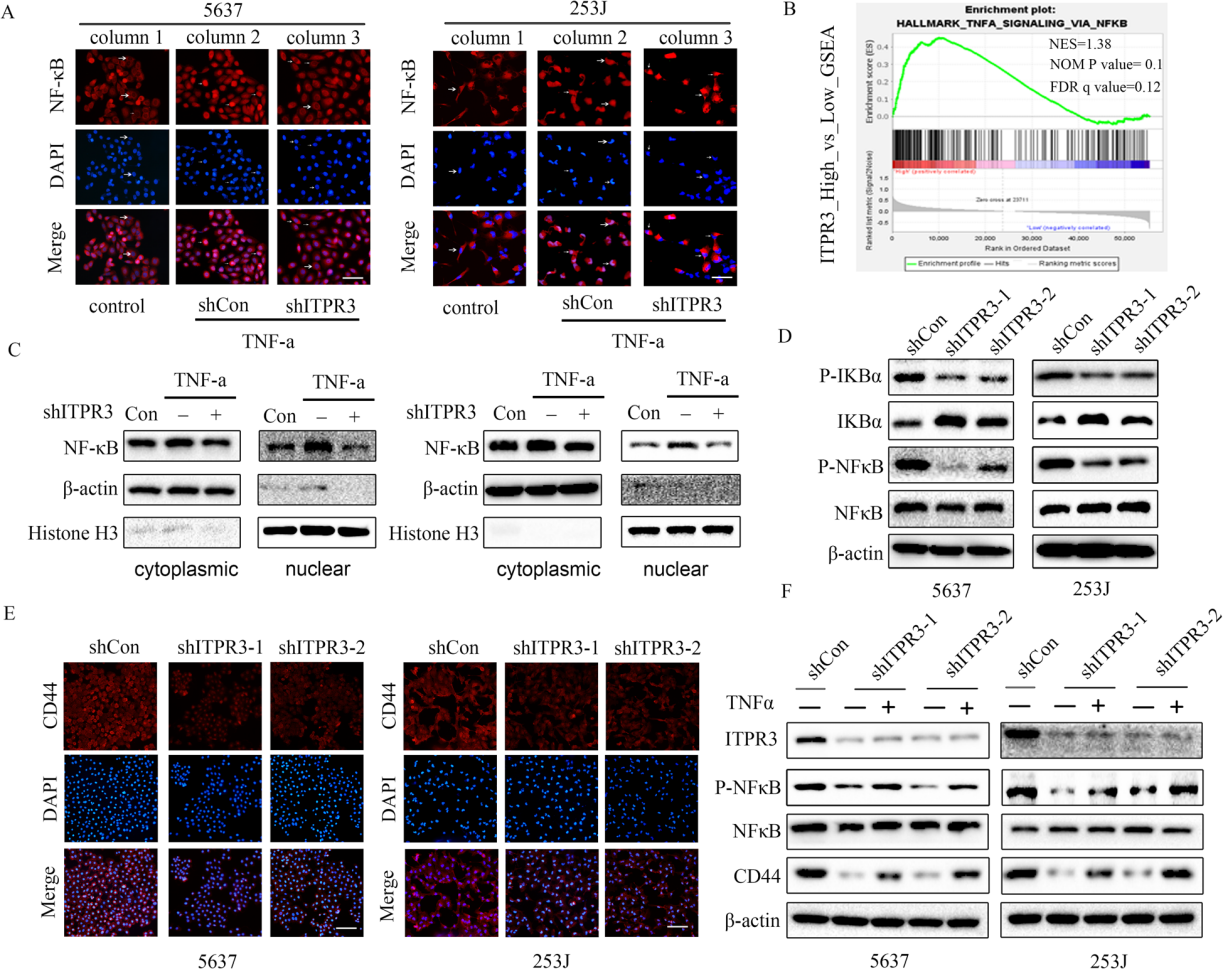
ITPR3-mediated NF-ĸB/CD44 signaling in BCa cells. **a** Immunofluorescence indicating the nuclear translocation of NF-κB (P65) induced by TNFα (10 ng/mL) (column 1 vs. column 2), which can be inhibited by ITPR3 knockdown (column 2 vs. column 3). Bar: 50 μm, arrows: NF-κB nuclear translocated cells. **b** GSEA results showed that “HALLMARK_TNFA_SIGNALING_VIA_NFKB” was upregulated and responded to high ITPR3 expression. **c** The expression level of NF-κB in nuclear and cytosolic fractions was analyzed by western blotting. Histone H3 and β-actin were used as the loading controls for the nucleus and cytosol, respectively. **d** We examined the NF-κB pathway-related proteins P-NF-κB, NF-κB, P-IKBa, and IKBα by western blotting with specific antibodies in 5637 and 253 J cells transfected with ITPR3 shRNA or shCon. β-actin served as an internal control. **e** CD44 expression was analyzed by immunofluorescence in 5637 and 253 J cells transfected with ITPR3 shRNA or shCon. Scale bars, 100 μm. **f** western blot analysis of ITPR3, CD44, NF-κB, and P- NF-κB in 5637 and 253 J cells treated with TNFα for 24 h after transfection with ITPR3 shRNA or shCon. β-actin was used as a loading control

NF-κB signaling plays a vital role in cancer initiation and progression and is abnormally activated in various cancers [23]. CD44, which is a cell surface glycoprotein and a CSC marker in BCa cells, is involved in various cellular processes, including cell adhesion, migration, and proliferation. However, the relationship and potential interaction between the two molecules in bladder cancer are still unknown. As shown in Fig. 6, we demonstrated that ITPR3 knockdown suppressed cancer stemness by decreasing the CD44 protein level. A similar result was also observed in the immunofluorescence assay for 5637 and 253 J cells (Fig. 7e). As shown in Fig. 7a, ITPR3 knockdown blocked the translocation of NF-κB (P65) from the cytoplasm to the nucleus induced by TNFα in 5637 and 253 J cells. Similar results were also observed in the western blot assay (Fig. 7c). Meanwhile, GSEA analysis was conducted and indicated that 41/50 gene sets were upregulated in the ITPR3 high-expression group. Coincidentally, “HALLMARK_TNFA_SIGNALING_VIA_NFKB” was also found to be upregulated when ITPR3 was highly expressed (Fig. 7b). All the results indicated that ITPR3 loss suppressed the nuclear translocation of NF-κB. Western blot analysis also showed that ITPR3 knockdown inactivated the NF-κB signaling pathway by decreasing the expression of P-IKBa and P-NFκB while increasing the expression of IKBa, which is an inhibitory factor of NF-κB (Fig. 7d). Previous studies have found that NF-κB might be upstream in the regulation of the expression of CD44 in other types of cancer [24, 25]. Therefore, we will focus on whether and how the two molecules interact in bladder cancer. To investigate the role of the NF-κB signaling pathway in regulating CD44 expression in BCa cells, bladder cancer cells were treated with TNFα (an activator of NF-κB signaling) after ITPR3 knockdown, and we found that TNFα reversed the expression of P-NFκB and CD44 simultaneously suppressed by ITPR3 knockdown, which indicated that activation of the NF-κB signaling pathway antagonized the low expression of CD44 caused by ITPR3 knockdown and that NF-κB functioned upstream of CD44 in BCa cells (Fig. 7f). In brief, these results suggested that ITPR3 mediated the NF-ĸB/CD44 signaling pathway in BCa cells.

### ITPR3 regulates proliferation and stemness via the NF-ĸB/CD44 signaling pathway in BCa

**Fig. 8:**
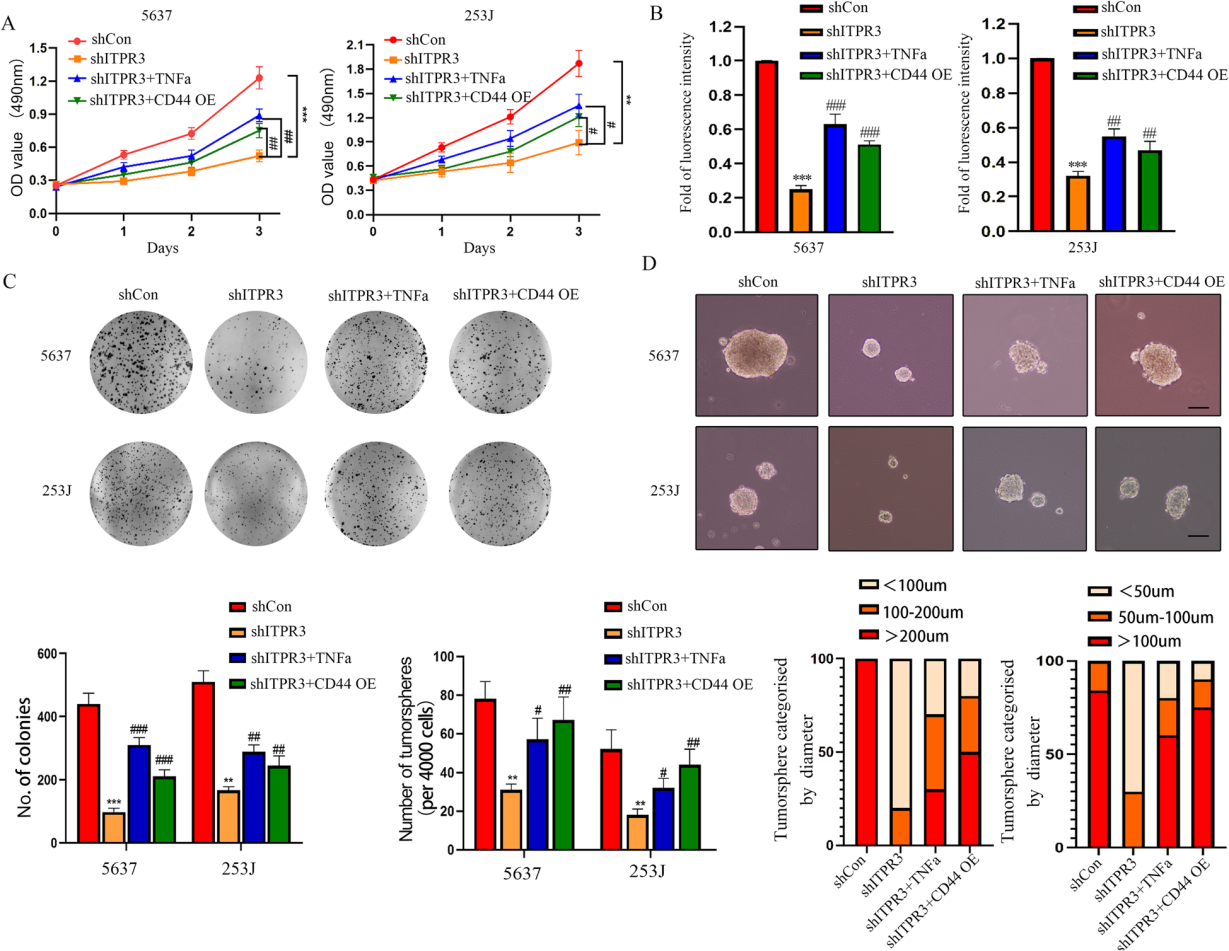
ITPR3 regulates proliferation and stemness via the NF-ĸB/CD44 signaling pathway in BCa. **a** An MTT assay was conducted to evaluate the viability of 5637 and 253 J shITRR3 or shCon cells after treatment with TNFα or overexpression of CD44. **b** An EdU staining assay was conducted to assess the proliferation ability of 5637 and 253 J shITRR3 or shCon cells treated as described above. **c** Colony formation assays were also performed in 5637 and 253 J shITRR3 or shCon cells treated as described above. Quantification analysis is shown below. **d** The cancer stemness was evaluated by a tumorsphere formation assay in 5637 and 253 J shITRR3 or shCon cells treated as described above. The scale bar represents 100 μm. Quantification analysis is shown below. ^*^p < 0.05, ^**^p < 0.01; ^***^p < 0.001 versus the scrambled shRNA (shCon) group, ^#^ *p* < 0.05, ^##^ *p* < 0.01, ^###^ *p* < 0.001 versus the ITPR3 shRNA group. CD44 OE: CD44 overexpression. shCon: Control shRNA

**Fig. 9:**
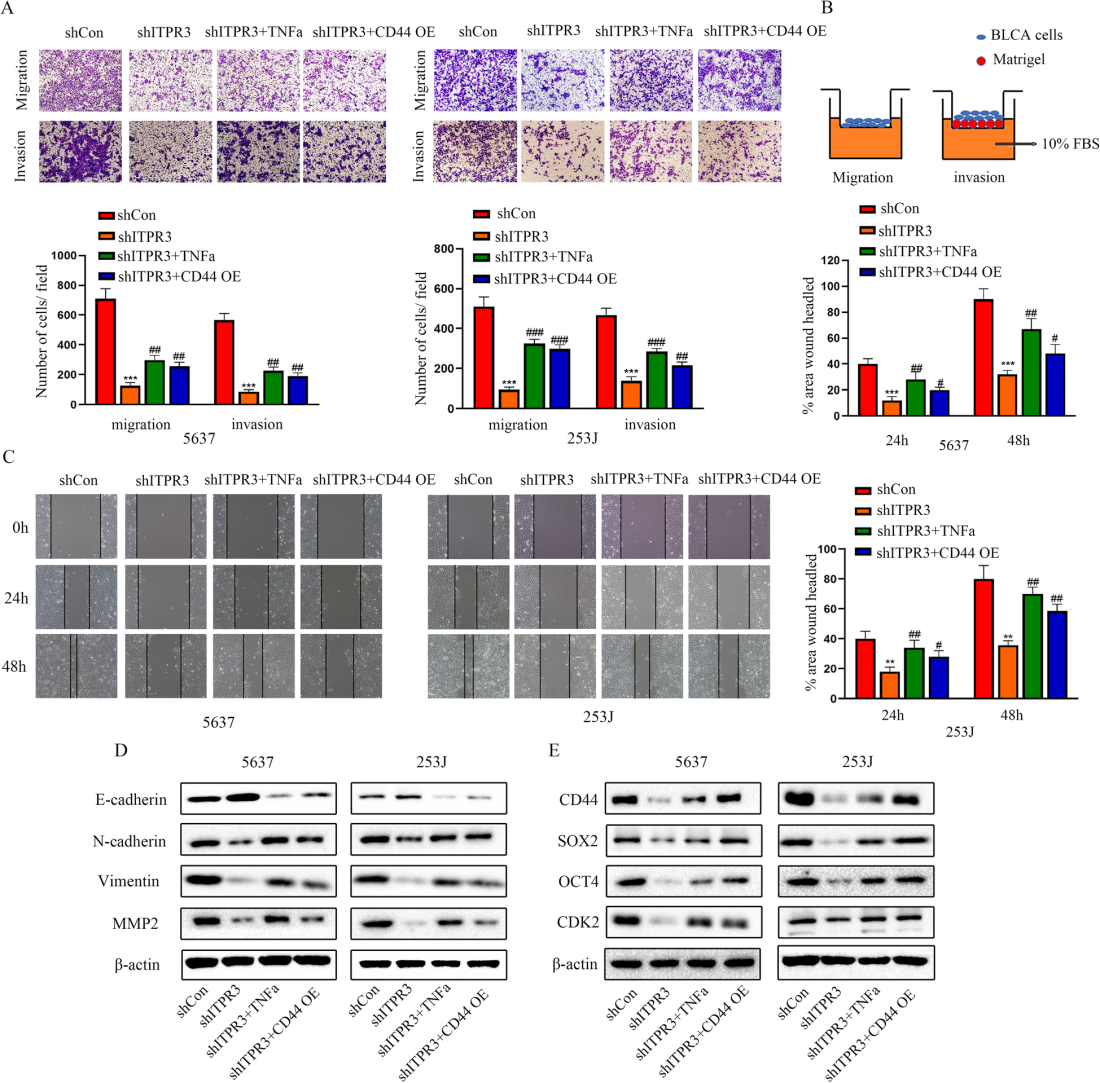
ITPR3 regulates EMT via the NF-ĸB/CD44 signaling pathway in BCa. **a** The migration and invasion abilities were analyzed by a transwell Boyden assay with or without Matrigel in 5637 and 253 J shITPR3 or shCon cells after treatment with TNFα or overexpression of CD44. Quantification analysis is shown below. **b** The schematic diagram of transwell assay. **c** A wound healing assay was also conducted as described above. Quantification analysis is shown on the right. **d** The gene expression of E-cadherin, N-cadherin, Vimentin and MMP2 was detected by a western blot assay in 5637 and 253 J cells after treatment as described above. β-actin served as an internal control. **e** The gene expression of CD44, OCT4, SOX2 and CDK2 was detected by a western blot assay in 5637 and 253 J cells after treatment as described above. β-actin served as a loading control. ^**^p < 0.01; ^***^p < 0.001 versus the scrambled shRNA (shCon) group, ^#^ *p* < 0.05, ^##^ *p* < 0.01, ^###^ p < 0.001 versus the ITPR3 shRNA group. CD44 OE: CD44 overexpression. shCon: Control shRNA

From the results above, we know that ITPR3 can regulate the NF-ĸB/CD44 signaling pathway in BCa. Next, it is necessary to dissect the mechanisms of NF-ĸB/CD44 signaling in regulating the malignant progression of bladder cancer. The key role of CD44 in maintaining the cancer stemness of BCa cells is inseparable from tumor growth, distant metastasis, and drug resistance. We established stable subcellular lines with ectopic CD44 overexpression in 5637 and 253 J shITPR3 cells. Here, we hypothesized that increased CD44 expression levels and activation of the NF-κB signaling pathway may be involved in ITPR3-promoted BCa growth and stemness. As is shown in Fig. 8a, Both TNFα treatment and CD44 overexpression partly reversed the proliferation inhibition induced by ITPR3 knockdown. Similar results were also observed in the EdU assay and colony formation assay (Fig. 8b, c). As expected, TNFα treatment and CD44 overexpression also significantly potentiated the CSC phenotype of BCa cells suppressed by ITPR3 knockdown (Fig. 8d). Consistently, western blotting data clearly showed that specific markers of proliferation and CSCs, such as CD44, SOX2, OCT4, and CDK2, were also upregulated after treatment as described above (Fig. 9e). These results collectively indicated that the oncogenic property of ITPR3 was mediated by the activation of the NF-ĸB/CD44 signaling pathway.

### ITPR3 regulates EMT via the NF-ĸB/CD44 signaling pathway in BCa

Recent studies have reported that EMT and CSCs always appear coincidently, and EMT has been identified as a critical regulator of the CSC phenotype in tumors [26]. CSCs can accelerate the EMT process, thus leading to distant metastasis [27]. Hence, a greater understanding of the interaction between EMT and CSCs will contribute to improvements in clinical treatment strategies. Transwell assays were conducted to evaluate the migration and invasiveness of 5637 and 253 shITPR3 cells after treatment with TNFα or ectopic CD44 overexpression, and a schematic diagram of the Transwell assay is shown in Fig. 9b. As is shown in Fig. 9a, both TNFα treatment and CD44 overexpression partly reversed the migration and invasiveness inhibition induced by ITPR3 knockdown, and similar results were also observed in the wound healing assay (Fig. 9c). Consistent with the results observed above, western blot assays showed that N-cadherin, vimentin and MMP2 were upregulated while E-cadherin was downregulated in 5637 and 253 J shITPR3 cells after TNFα and ectopic CD44 overexpression treatment (Fig. 9d). These results indicated that ITPR3 regulated EMT via the NF-ĸB/CD44 signaling pathway in BCa cells.

### ITPR3 knockdown attenuated the tumorigenicity of BCa in vivo

**Fig. 10:**
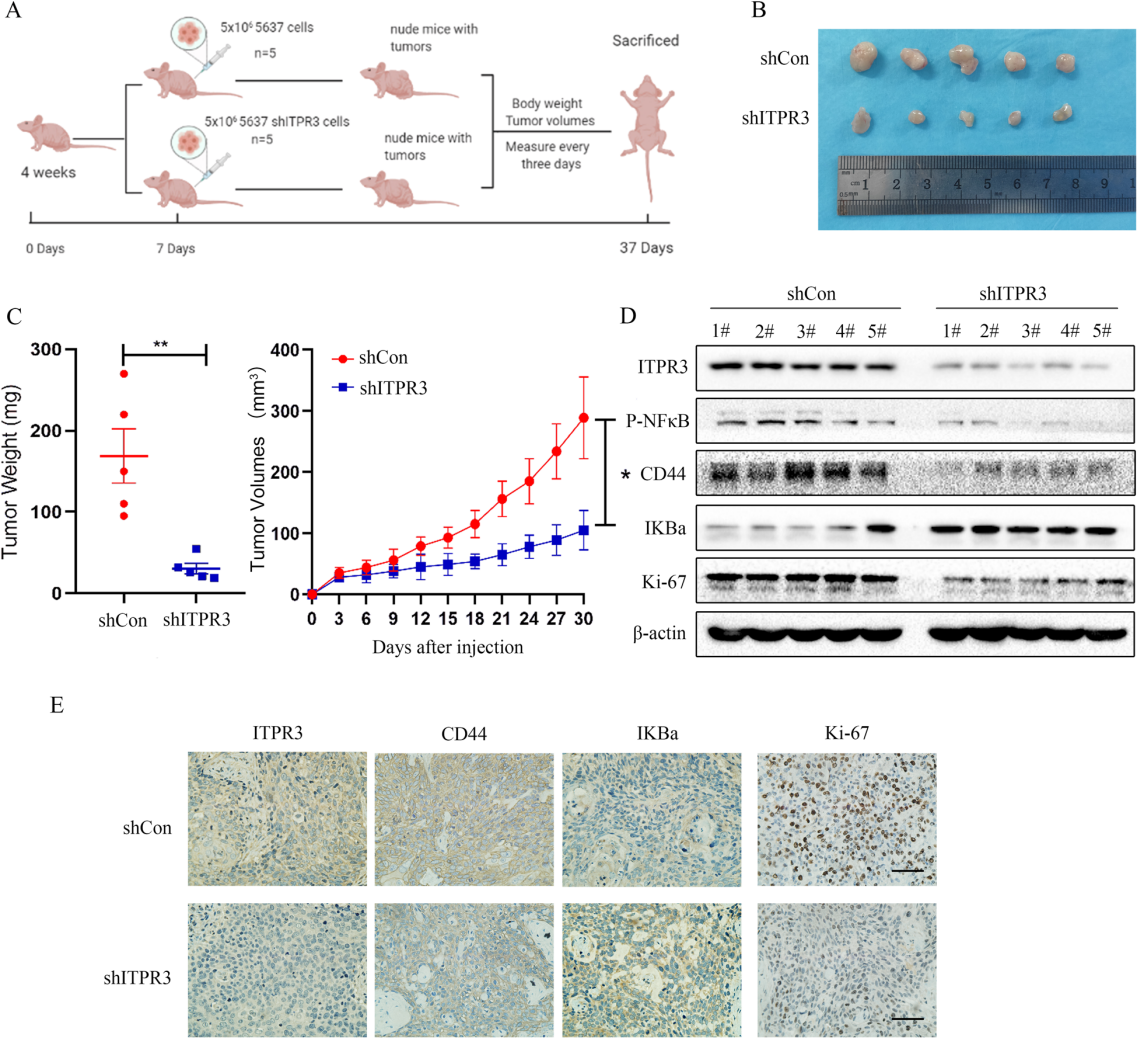
ITPR3 knockdown attenuated the tumorigenicity of BCa in vivo. **a** Schematic diagram of the subcutaneously implanted tumor model in nude mice. **b** Tumors were obtained from nude mice in the shITPR3 group and shCon groups, and representative images are shown in the right panel. **c** The wet weights of tumors in the shITPR3 and shCon groups were measured and compared after the mice were sacrificed at the end of the experiment. Meanwhile, the tumor volume changes in the nude mice were measured every three days. **d** Protein expression levels of ITPR3, P-NF-κB, IKBa, Ki-67, and CD44 were detected by a western blot assay, and β-actin was used as an internal control. **e** Immunohistochemistry was used to analyze the expression of ITPR3, CD44, Ki-67 and IKBa. The scale bar represents 50 μm. ^*^P < 0.05, ^**^P < 0.01

The tumorigenicity of ITPR3 in vivo was validated in a xenograft model. We injected ITPR3-depleted or control 5637 cells into nude mice. Tumor volumes and body weights were measured every three days within 30 days, and tumors were harvested after the mice were sacrificed (Fig. 10a). Representative images of tumors are presented in Fig. 10b. As shown in Fig. 10c, the xenograft tumors grew more slowly after ITPR3 knockdown compared with the control, and the tumor weight was much lighter in the ITPR3-depleted group. Western blot assays were also conducted to detect the expression of related genes. The results showed that ITPR3, P-NF-κB, CD44, and Ki-67 were downregulated, while IKBa was upregulated after ITPR3 knockdown (Fig. 10d). Moreover, immunohistochemistry staining showed that ITPR3, CD44 and Ki-67 were weaker, while IKBa was stronger in ITPR3-silenced tumor tissues (Fig. 10e). These results suggested that ITPR3 knockdown attenuated the tumorigenicity of BCa in vivo.

### ITPR3 knockdown suppressed BCa cell distant metastasis in vivo

**Fig. 11:**
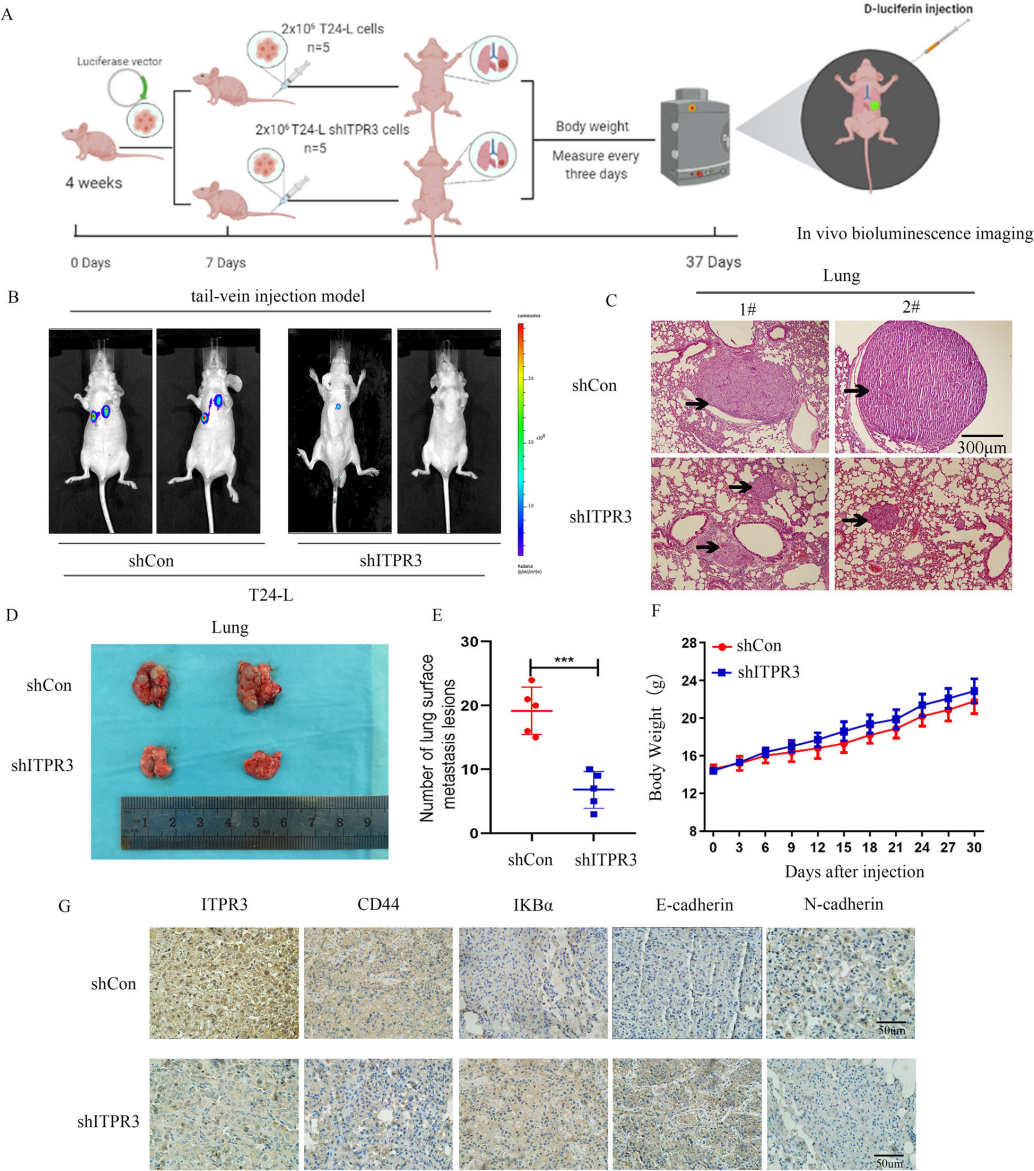
ITPR3 knockdown suppressed BCa cell distant metastasis in vivo. **a** Schematic diagram of the tail vein injection metastasis model in nude mice. **b** Representative bioluminescence images from the shITPR3 and shCon groups are shown in the left panel. **c** The histological changes detected by HE staining of the lungs from the shITPR3 and shCon groups are shown in the right panel. Arrows: foci of lung metastasis. **d** The general appearance of the lungs from the shITPR3 and shCon groups is presented in the left panel. **e** The number of lung surface metastatic lesions from the shITPR3 and shCon groups was also calculated. **f** The body weight changes in the shITPR3 and shCon groups were measured and recorded every three days. **g** Representative images of immunohistochemistry staining of ITPR3, CD44, E-cadherin, N-cadherin and IKBa are presented below. The scale bar represents 50 μm

The T24-L subcell line tagged with luciferase was used to establish the tail vein injection metastasis model, which was used to evaluate the potential role of ITPR3 in BCa distant metastasis. A schematic diagram of the experimental procedure is shown in Fig. 11a, which was also described in our previous study [12]. Bioluminescence imaging (BLI) showed that lung metastasis was almost abolished after ITPR3 knockdown compared with the control (Fig. 11b). Similar results were also observed by hematoxylin-eosin staining, in which the formation of lung metastatic foci was significantly suppressed in shITPR3 T24-L cells compared with control shRNA cells (Fig. 11c). Lungs were removed to observe the number of lung surface metastatic foci, and ITPR3 depletion obviously reduced the metastatic nodules in mouse lungs compared with the control (Fig. 11d, e). Meanwhile, there was no distinct change in body weight between the shITPR3 T24-L cells and shCon T24-L cells (Fig. 11f). IHC (immunohistochemistry) also showed that ITPR3, CD44, and N-cadherin were weaker, while IKBa and E-cadherin were stronger in the ITPR3-depletion group (Fig. 11g). These results indicated that ITPR3 knockdown inhibited BCa metastasis in vivo.

## Discussion

Patients with metastatic BCa usually suffer a poor prognosis due to a lack of an effective treatment strategy [28]. In BCa, distant metastasis, especially lung metastasis, and chemoresistance are the two leading causes of death. Therefore, there is an urgent need to explore more molecular mechanisms of BCa malignant progression.

ITPRs are ubiquitous Ca2+ channels located at mitochondria/endoplasmic reticulum (ER) contact sites and can interact with GRP75 to accelerate the transmission of calcium from the lumen of the ER directly into mitochondria [29]. Intracellular calcium (Ca2+) signaling pathways mediate various cellular processes, including tumor development and proliferation, gene transcription, cell migration and invasion, cell apoptosis and necrosis [7]. ITPR3 accounts for 90% of ITPRs, while ITPR1 and ITPR2 constitute approximately 10% [30]. Previous studies have failed to address the specific mechanism of ITPR3 oncogenic roles in a variety of cancers, which attracts our attention. Several investigators have reported that the loss of ITPR3 can impair mitochondrial calcium signaling and reduce apoptosis, thus leading to premalignant conditions in various cancers, such as melanoma and mesothelioma [31]. While in prostate cancer, ITPR3 can limit tumor growth by regulating Ca2 + −dependent apoptosis, in which the more ITPR3 is degraded, the less apoptosis occurs [32]. In colon cancer, increased ITPR3 expression is related to increased metastasis and decreased patient survival, and increased ITPR3 can lead to reduced apoptosis or vice versa [33]. A similar phenomenon was also observed in hepatocellular carcinoma and cholangiocarcinoma [7, 9]. It is worth noting that we demonstrated for the first time that ITPR3 was overexpressed and served as an oncogene in bladder cancer. Previous studies have confirmed that ITPR3 can be suppressed by transcription factors or miRNAs [34, 35], while the factors causing the overexpression of ITPR3 are not yet clear. Methylation is an important modification of protein and nucleic acid, which can regulate gene expression and shutdown. Meanwhile, Methylation is closely related to cancer, aging, Alzheimer’s disease, and many other diseases, and is also one of the important contents of epigenetics research [36]. Gene methylation is closely related to gene expression level and the stability of chromatin. In general, activation of proto-oncogenes is accompanied by demethylation of their promoters and demethylation of DNA is a rapid, independent, and distinct process from cell division. Both hypermethylation of tumor suppressor genes as well as hypomethylation of oncogenes genes are characteristic of cancer cells. As DNA methylation is reversible, Demethylation may play an important role in the occurrence and development of tumors [37]. Bioinformatic analysis has confirmed that there are four CpG islands in the ITPR3 promoter region, including three CpG islands longer than 200 bp. BSP also confirmed that the methylation of the ITPR3 promoter in the normal bladder cell line SVHUC-1 was higher than that in the bladder cancer cell lines (5637 and 253 J) at 43 different CpG sites in 10 clones, and there was also a negative correlation between the mRNA level and methylation level in ITPR3 in bladder cancer. 5-Aza, as a DNA methyltransferase inhibitor, can also upregulate the expression of ITPR3 in SV-HUC-1 cells, which indicates that the expression of ITPR3 could be affected by the methylation level in the gene promoter. Therefore, we can conclude that demethylation of the ITPR3 promoter is responsible for its high expression in bladder cancer. From our work in this research, we demonstrated the oncogenic role of ITPR3 in bladder cancer in contrast to the roles of ITPR1 and ITPR2, whose proapoptotic effect was already described [6]. We also found that ITPR1 was expressed at low levels in bladder cancer, while there was no obvious difference in ITPR2 expression compared with normal expression which was consistent with the result of western-blot. Considering the contradictory roles of ITPR1, ITPR2 and ITPR3 in tumors, a western blot assay was conducted to assess the expression of ITPR1 and ITPR2 when ITPR3 was knocked down. The results showed that ITPR3 depletion upregulated the expression of ITPR1 but had no effect on ITPR2 (Supplementary Fig. 2). What is the mechanism by which ITPR3 realizes its oncogenic effect? From the results above, we hypothesize that the oncogenic effect of ITPR3 is partly attributed to the inhibition of ITPR1 and that the ratio of ITPR3/ITPR1 may determine the destiny of cancer cells which is consistent with the conjectures in the previous research [6].

It is generally accepted that CSCs (cancer stem cells) are defined as a subpopulation of highly tumorigenic cancer cells with self-renewal activity, and they have been reported to exist in various types of cancer [38]. Cancer stem cells (CSCs) mediate tumor initiation and maintenance, which can contribute to distant metastasis and a poor prognosis. EMT was reported to exist in the cancer stem cell population and is driven by CSCs [39]. Therefore, CSC-targeting therapy has already become a new direction for the treatment of cancer in the future, especially metastatic cancer. As shown in Supplementary Fig. 3, ITPR3 and CSC markers such as CD44, SOX2 and OCT4 were enriched in BCa stem cells, which indicated that ITPR3 might play a vital role in maintaining the cancer stemness phenotype. We demonstrated that ITPR3 depletion suppressed cancer cell proliferation, migration, invasion and stemness by inhibiting the expression of CD44, which is the main cancer stem cell marker in bladder cancer [40].

NF-κB (nuclear factor (NF)-kappa B) serves as a transcription factor and has been reported to activate various genes, including inflammatory cytokines and oncogenes, which suppress the apoptosis of cancer cells. NF-κB is usually abnormally activated in cancers and plays a vital role in promoting cancer development and progression, in addition to hindering the effectiveness of chemo- and radiation therapies [41]. In this research, GSEA was used to verify that “HALLMARK_TNFA_SIGNALING_VIA_NFKB” was upregulated when ITPR3 was highly expressed. The heat map and other gene sets are also shown in Supplementary Fig. 4. ITPR3, as an oncogene, plays a critical role in promoting proliferation and metastasis and maintaining tumor stemness depending on NF-κB activation. Meanwhile, CD44 expression was suppressed by the inactivation of NF-κB signaling induced by ITPR3 knockdown. Nevertheless, how ITPR3 influence Ca + signaling and the relationship between the Ca + signaling and NF-κB signaling should be further analyzed.

## Conclusion

**Fig. 12:**
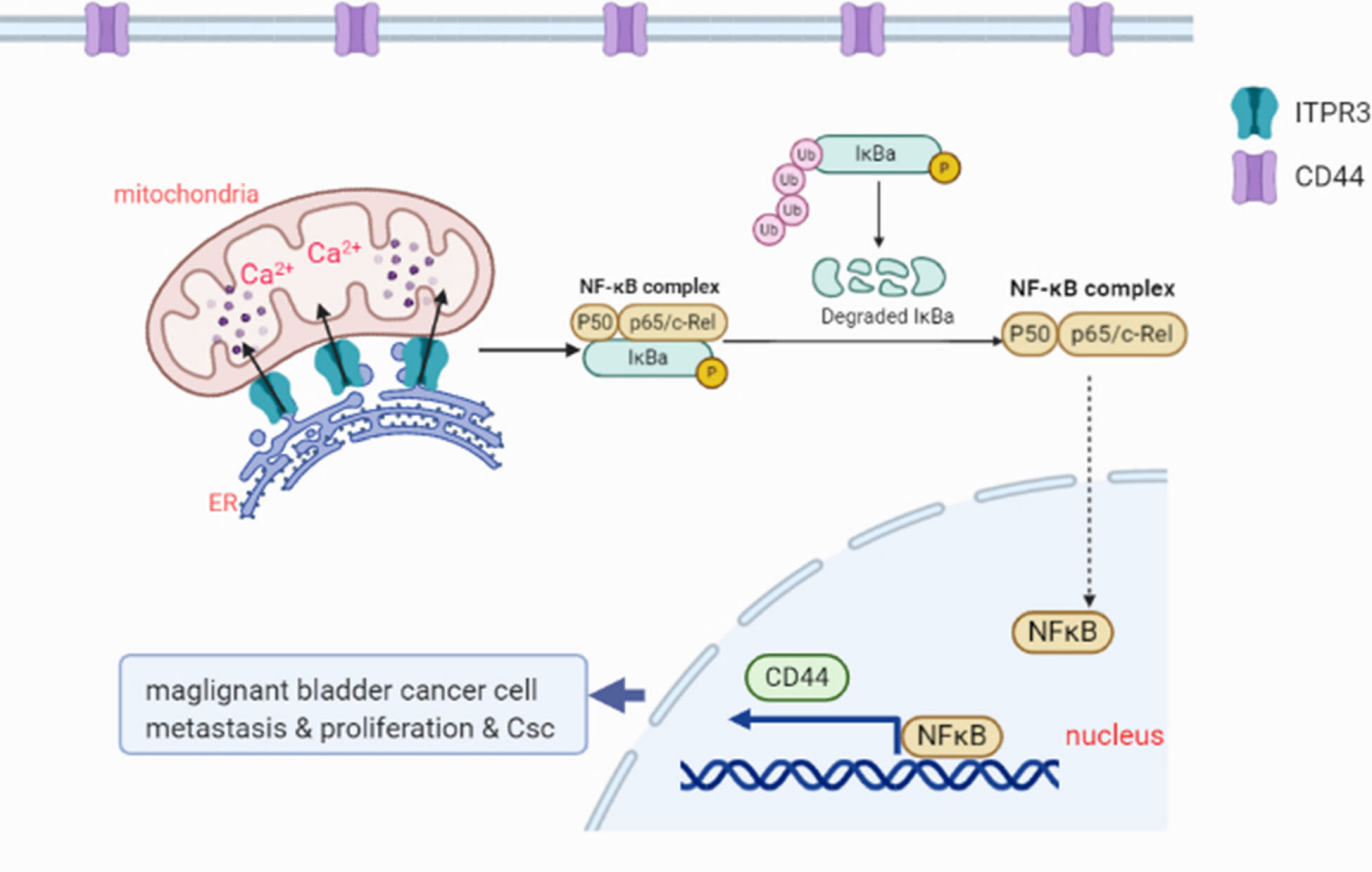
An illustration of how ITPR3 facilitates tumor growth, metastasis and stemness by inducing the NF-ĸB/CD44 pathway

In conclusion, we demonstrated in this research that ITPR3, as an oncogenic gene, promoted the proliferation, metastasis and stemness of bladder cancer through the NF-κB/CD44 signaling pathway. CD44, as a stem cell marker of bladder cancer could be regulated by NF-κB signaling pathway which played an important role in bladder cancer. Taken together, ITPR3 could be a novel prognostic marker and drug target for BCa, especially metastatic BCa (Fig. 12).

## Supplementary Information

**Additional file 1: Supplementary Figure 1**. Bioinformatics analysis of ITPR3 expression in bladder cancer and correlation between ITPR3 and overall survival in BCa patients. (A) ITPR3 mRNA expression in bladder cancer tissue and normal adjacent tissue from the GEPIA website based on TCGA database. (B, C) ITPR3 mRNA expression in bladder cancer tissues and adjacent tissues from the TCGA dataset and GEO (GSE3167, GDS183) dataset. (D) ITPR3 is significantly upregulated in bladder cancer (left), as is ITPR3 mRNA expression in multiple kinds of cancers (right), which was analyzed from the Oncomine database. (E) ITPR3 was overexpressed in bladder cancer compared with normal tissues in Sanchez-Carbayo’s research from the Oncomine dataset. (F) Kaplan-Meier analysis of overall survival (OS) and disease-free survival (DFS) in BCa patients with high (*n* = 201) and low (n = 201) ITPR3 mRNA expression from TCGA database.

**Additional file 2: Supplementary Figure 2**. The expression of ITPRs in BCa and the relationship between ITPR3 and ITPR1 or ITPR2. (A) Analysis of ITPR1, ITPR2, and ITPR3 mRNA expression in bladder cancer and normal tissues from the TCGA dataset. (B) ITPR1, ITPR2, and ITPR3 mRNA expression levels in SV-HUC-1, 5637, and 253 J cells were detected by a qRT-PCR assay. (C) ITPR1, ITPR2, and ITPR3 protein levels were examined by a western blot assay in 5637 cells transfected with shITPR3 or shCon.

**Additional file 3: Supplementary Figure 3**. ITPR3 was enriched in the CSC. (A) The morphological differences of 5637 and CSCs derived from 5637 cells were captured by contrast microscopy. (B) 253 J was also observed in the same way. (C) The expression of ITPR3 and cancer stem cell markers such as CD44, SOX2, and OCT4 was detected by western blot assay in 5637 and CSCs derived from 5637 cells. (D) The expression of ITPR3 and cancer stem cell markers such as CD44, SOX2, and OCT4 was detected by a western blot assay in 253 J and CSCs derived from 253 J cells.

**Additional file 4: Supplementary Figure 4**. GSEA analysis of ITPR3 in the TCGA dataset and significantly changed cell signaling pathways in 50 hallmark gene sets by ITPR3 expression. (A) Heat map of the top 100 genes upregulated or repressed in the ITPR3 high-expression and ITPR3 low-expression BCa patient groups. (B) The significantly changed cell signaling pathways in 50 hallmark gene sets from GSEA analysis of ITPR3 expression in the BCa TCGA dataset.

## Data Availability

The datasets generated/analyzed during the current study are available.

## Abbreviations

BCa: Bladder cancer
CSC: Cancer stem cell
NF-κB: Nuclear factor-kappaB
IKBα: NF-kappa-B inhibitor alpha
ITPR3: Inositol 1,4,5-Trisphosphate Receptor type 3
EMT: Epithelial-mesenchymal transition
TGF-β: Transforming growth factor-β
TNFα: Tumor necrosis factor α
MTT: 3-(4,5-dimethylthiazol-2-yl)-2,5-diphenyl tetrazolium bromide
5-Aza: 5-Aza-2′-deoxycytidine
HE: Hematoxylin-eosin
DMEM: Dulbecco’s modified Eagle medium
FBS: Fetal bovine serum
BSP: Bisulfite sequencing PCR
T24-L: T24-T-Luciferase-Lung subcell line
NMIBC: Non-muscle invasive bladder cancer
MIBC: Muscle invasive bladder cancer
